# Fast Evaluation and Comparison of the Energy Performances of Elastomers from Relative Energy Stored Identification under Mechanical Loadings

**DOI:** 10.3390/polym14030412

**Published:** 2022-01-20

**Authors:** Jean-Benoît Le Cam

**Affiliations:** Institut de Physique UMR 6251 CNRS de Rennes 1, Campus de Beaulieu, Université de Rennes 1, Bât. 10B, CEDEX, 35042 Rennes, France; jean-benoit.lecam@univ-rennes1.fr

**Keywords:** elastomer, fast characterization, energy stored and released, heat source reconstruction, intrinsic dissipation, infrared thermography

## Abstract

The way in which elastomers use mechanical energy to deform provides information about their mechanical performance in situations that require substantial characterization in terms of test time and cost. This is especially true since it is usually necessary to explore many chemical compositions to obtain the most relevant one. This paper presents a simple and fast approach to characterizing the mechanical and energy behavior of elastomers, that is, how they use the mechanical energy brought to them. The methodology consists of performing one uniaxial cyclic tensile test with a simultaneous temperature measurement. The temperature measurement at the specimen surface is processed with the heat diffusion equation to reconstruct the heat source fields, which in fact amounts to surface calorimetry. Then, the part of the energy involved in the mechanical hysteresis loop that is not converted into heat can be identified and a quantity $\gamma_{se}$ is introduced for evaluating the energy performance of the materials. This quantity is defined as an energy ratio and assesses the ability of the material to store and release a certain amount of mechanical energy through reversible microstructure changes. Therefore, it quantifies the relative energy that is not used to damage the material, for example to propagate cracks, and that is not dissipated as heat. In this paper, different crystallizable materials have been considered, filled and unfilled. This approach opens many perspectives to discriminate, in an accelerated way, the factors affecting these energetic performances of elastomers, at the first order are obviously the formulation, the aging and the mechanical loading. In addition, such an approach is well adapted to better characterize the elastocaloric effects in elastomeric materials.

## 1. Introduction

Elastomers are widely used in many industries, such as automotive, nuclear or civil engineering, for their high deformability, high damping and, for some of them, their high fatigue resistance. As for most of the engineering materials, their design requires substantial characterization time and cost, typically for fatigue [1,2,3,4,5,6,7], crack propagation [8,9,10], aging [11,12,13] and damping [14], non-exhaustively. At a time when many sectors that use elastomeric materials are undergoing deep technological changes, such as health with implantable medical devices or mobility with the desire to decarbonize transportation, it is necessary to develop new materials and new technological solutions that must be evaluated in an increasingly limited time and at a low cost. In addition, crystallizing elastomers are increasingly studied for their elastocaloric properties [15,16,17] and criteria are needed to compare their energy performance. This is why the development of fast characterization methods for rubbers, allowing a classification of the desired performances, however basic, is today a major challenge in most industries.

What makes elastomers attractive for many applications is that their mechanical response exhibits a hysteresis loop. The mechanical hysteresis is generally obtained by adding fillers in the rubber matrix and/or strain-induced crystallization (SIC) and melting. Classically, the mechanical energy involved in the hysteresis loop is assumed to be mainly dissipated into heat. Nevertheless, several observations question this assumption:

- The mechanical response of some elastomers exhibits a hysteresis loop only when strain-induced crystallization (SIC) occurs, typically in case of unfilled natural rubber (NR) [18]. In this case, no self-heating accompanies the mechanical cycles, which indicates that SIC does not induce or has very little viscosity. This was first intuited by Clark [19] and confirmed by [20,21,22];
- The mechanical hysteresis can be not time-dependent [23,24,25,26]. This is not expected for viscous materials;
- If all the energy contained in the hysteresis loop were due to viscosity, the self-heating would be much higher than that observed experimentally, especially under repeated cycles.

One can therefore wonder about the nature and the time dependency of the phenomena involved in the formation of the hysteresis loop and about the real contribution of the intrinsic dissipation to it. To go further in the discussion, it is necessary to recall that in addition to the intrinsic dissipation two other factors can affect to the mechanical hysteresis:

(a) The thermal dissipation (under non adiabatic test conditions). If heat is exchanged with the specimen’s outside, then a hysteresis loop in the stretch-stress relationship can theoretically form, the current temperature appearing in the elastic coupling. In most of the homogeneous tests (in terms of heat source field, see Section 2) performed, considering the thermal properties of elastomers and the loading rate relatively high, the thermal dissipation does not contribute significantly to the mechanical hysteresis;
(b) The change in microstructure. In this case, all the work done to the system is not measured as an apparent temperature change (see for instance the recent studies by [27] on polyurea who concluded that a significant part of the mechanical energy is used by the material to reorganize or by Le Cam [22] who demonstrated that the mechanical hysteresis of the unfilled natural rubber he studied was entirely due to the difference in kinetics between crystallization and crystallite melting).

Thus, of the many phenomena that can contribute to the hysteresis loop, some do not appear to dissipate energy as heat. This is in fact what is assessed with self-heating tests for evaluating the fatigue properties [28]. Therefore, the level of self-heating or more precisely intrinsic dissipation must be put into perspective in relation to the energy rate involved in the hysteresis loop. Even more interesting is the part of the mechanical energy brought that is stored and released by reversible microstructure changes, which is not dissipated as heat or used to damage the material, for example to propagate cracks. In the extreme, the mechanical response of an unfilled natural rubber exhibits a hysteresis loop as soon as it is crystallizing, while no (or minimally) energy is converted as heat [22].

Considering that contributions to the hysteresis loop change when one chemical formulation is substituted to another one (see for example [29]), or when one or more ingredients of a chemical formulation is changed to improve a given property (see, for example, [30,31,32,33]), the present study assumes that identifying the energy contributions and associated phenomena involved in the mechanical hysteresis is a reliable indicator of the material’s performances.

Such analysis echoes the pioneering work by [34,35] who measured the latent energy remaining after cold working in a metal under quasi-static monotonous loadings. Today, the fraction of the anelastic deformation energy rate irreversibly converted into heat is studied through the Taylor–Quinney ratio [36,37,38,39,40]. Polymers have then benefited from this approach in the Rittel’s group [41,42] and the Chrysochoos’s group [43,44]. Concerning elastomers, only four recent studies investigate the energetic behavior and the energy storage during deformation [22,45,46,47]. From these studies, the energy storage in different types of elastomers, filled and unfilled, crystallizing or not, can be discussed. The main difference with the pre-mentioned materials is that the energy stored elastically with a given kinetics is released within the same mechanical cycle, but with a different kinetics. This energy serves for reversible changes in microstructure, typically when the crystallization—crystallite melting process occurs in natural rubbers [20,48]. Section 2 presents the thermodynamics framework for calorimetry under stretch from surface temperature measurements, the methodology for energy balances and the definition of the characteristic quantity giving the energy performance. Section 3 presents a typical experimental setup. Section 4 provides illustrations with different materials, inspired from three recent studies carried out in our group [22,45,47], which provides the first comparison of elastomers according to their energy performances. Concluding remarks close the paper.

## 2. Thermodynamic Framework

Identifying the phenomena involved in the mechanical hysteresis requires the calculation of several continuum quantities that are recalled in this section. The calculations are carried out in the case of the uniaxial tension.

### 2.1. Total Strain Energy Density and Energy Rate Involved in the Hysteresis Loop

The strain energy density $W_{strain}$ (in J/m${}^{3}$) is the energy brought mechanically to deform the material. It corresponds to the area under the load (unload) strain–stress curve and is calculated as follows:

(1) $$W_{strain}^{load}=\int_{load}\pi \;d\lambda \;\mathrm{and}\;W_{strain}^{unload}=\int_{unload}\pi \;d\lambda ,$$

where λ is the stretch defined as the ratio between current and initial lengths. π is the nominal stress, defined as the force per unit of initial (undeformed) surface. If the material’s behavior is purely elastic and if the test is carried out under adiabatic loading conditions, then the mechanical response obtained during a load-unload cycle is such that no hysteresis loop forms ($W_{strain}^{load}=W_{strain}^{unload}$). If a hysteresis loop forms, the mechanical energy dissipated over one cycle $W_{hyst}^{cycle}$ is defined as follows:

(2) $$W_{hyst}^{cycle}=W_{strain}^{load}-W_{strain}^{unload}.$$

From this energy, a quantity $P_{hyst}^{cycle}$ is calculated in W/m${}^{3}$. It is obtained by dividing $W_{hyst}^{cycle}$ by the cycle duration. It is therefore an energy density per time unit or an energy rate. It should be noted that when the mechanical cycle is also a thermodynamic cycle, the energy contained in the hysteresis loops is only due to intrinsic dissipation and thermomechanical coupling effects as long as the specific heat is assumed to be constant (further details are provided in [49,50]). This will be used in the following for carrying out the energy balances and to identify the intrinsic dissipation.

### 2.2. Heat Sources

During the mechanical cycle, the material produces and absorbs heat. Under non adiabatic conditions, corresponding temperature variations are influenced by heat diffusion effects. Therefore, temperature is not the relevant quantity to investigate the thermomechanical behavior of materials under non-adiabatic conditions, as most of classical mechanical tests are. This is the reason why the heat source (or the heat power density) is calculated from the heat diffusion equation and the temperature measurement (see [51,52] for further details). The heat source does not depend on the heat diffusion effects and is thus intrinsic to the thermomechanical behavior of materials. Considering the heat source field as homogeneous during uniaxial tensile loading, the formulation of the heat diffusion equation can be simplified as follows:

(3) $$\rho C\;\left(\dot{\theta }+\frac{\theta }{\tau }\right)=S,$$

where ρ is the initial density, *C* is the specific heat, θ is the temperature variation with respect to the equilibrium temperature $T^{ref}$ in the reference state, corresponding here to the undeformed state. The heat sources *S* can be divided into two terms that differ in nature:

- the intrinsic dissipation $D_{int}$: this positive quantity corresponds to the heat production due to mechanical irreversibilities during the deformation process, for instance viscosity or damage;
- the thermomechanical couplings $S_{tmc}$: they correspond to the couplings between the temperature and the state variables, and describe reversible deformation processes. Consequently, their integration with respect to time over one thermodynamical cycle is null.

Equation (3) is formulated in the case where the ambient temperature $T_{amb}$ is constant during the test. In case where changes in ambient temperature occur, the term $\frac{\theta }{\tau }$ has to be corrected accordingly as $\frac{T-T_{amb}}{\tau }$. τ is a parameter characterizing the heat exchanges between the specimen and its surroundings. It can be easily identified from a natural return to room temperature after a heating (or a cooling) for each testing configuration (machine used, environment, stretch level, etc). For instance, in cases where the material is beforehand heated, the exponential formulation of the temperature variation $\theta \left(t\right)=\theta_{0}e^{\frac{-(t-t_{0})}{\tau }}$ is used to determine τ, where *t* is the time in *s* and $\theta_{0}=\theta \left(t=t_{0}\right)$. Further details are provided in [18]. It should be noted that under uniaxial tension, τ depends on the stretch only (τ=τ(λ)) (It should be noted that in the case of heterogeneous strain fields, τ spatially varies according to the heterogeneous change in the thickness, that is, to the stretch and to the biaxiality ratio *B* (τ=τ(λ,B). Further information on the identification of a τ field is provided in [53]). Several strategies can be applied for determining τ(λ). Either it is evaluated for different stretch levels or by considering the material to be incompressible and the thickness changes to be proportional to $\lambda^{-\frac{1}{2}}$, so that $\tau =\tau \left(\lambda \right)=\tau_{0}\lambda^{-\frac{1}{2}}$. It should be noted that when the material is crystallizing, the second strategy is preferred. Indeed, a change in temperature of the material (heating or cooling) changes the crystallinity. Therefore, an additional heat production or absorption is obtained during the return at the initial crystallinity, which affects the temperature variation during the return at ambient temperature and, consequently, the identification of τ.

### 2.3. Identifying the Mean Intrinsic Dissipation

Integrating the heat source, that is, the left member of Equation (3), with respect to time over one thermodynamical cycle gives the energy density due to intrinsic dissipation, the integration of the thermomechanical couplings being null. This energy is then divided by the cycle duration to obtain the mean intrinsic dissipation ${\tilde{D}}_{int}$. This amounts to applying the following formula:

(4) $${\tilde{D}}_{int}=\frac{1}{t_{cycle}}\int_{cycle}S\;dt=\frac{1}{t_{cycle}}\int_{cycle}\left(D_{int}+S_{tmc}\right)\;dt=\frac{1}{t_{cycle}}\int_{cycle}D_{int}\;dt.$$

### 2.4. Energy Balance

Energy balance is carried out from both the mechanical and the calorific responses. The mechanical response provides the strain energy density and its rate $P_{hyst}^{cycle}$ involved in the hysteresis loop. The difference between $P_{hyst}^{cycle}$ and ${\tilde{D}}_{int}$ gives the rate of the energy stored and released due to microstructure changes at each cycle:

(5) $$P_{stored}^{cycle}=P_{hyst}^{cycle}-{\tilde{D}}_{int}.$$

To further discuss on the relative contribution of the energy stored in the hysteresis loop of rubbers, a ratio $\gamma_{se}$ has been proposed in our group [45,46]. It is written in terms of energy as follows:

(6) $$\gamma_{se}=\frac{W_{stored}^{cycle}}{W_{hyst}^{cycle}}$$

- if $\gamma_{se}$ tends to 0, no energy is stored during the deformation. The whole hysteresis loop is due to the intrinsic dissipation,
- if $\gamma_{se}$ tends to 1, the whole hysteresis loop is due to energy stored and no intrinsic dissipation is detected. This is typically the case in unfilled natural rubber [18], for which the energy stored in the crystallites is released with a different kinetics during their melting.

## 3. Overview of the Experimental Setups

Three different materials have been considered:

- an unfilled natural rubber, denoted U-NR and studied in [21]. Its chemical composition is given in Table 1;
- a carbon black filled natural rubber studied in [47], denoted F-NR. Its chemical composition is given in Table 1;
- an unfilled thermoplastic polyurethane, also crystallizable under tension, denoted TPU and studied in [45]. It is referred to as Irogran® A87H4615 TPU from the Huntsman corporation (The Woodlands, TX, USA). It is elaborated by reacting together a diisocyanate, a macro diol (long chain diol), which is a polyester in the present case, and a small molecule chain-extender (butane diol) [54].

**Figure 1:**
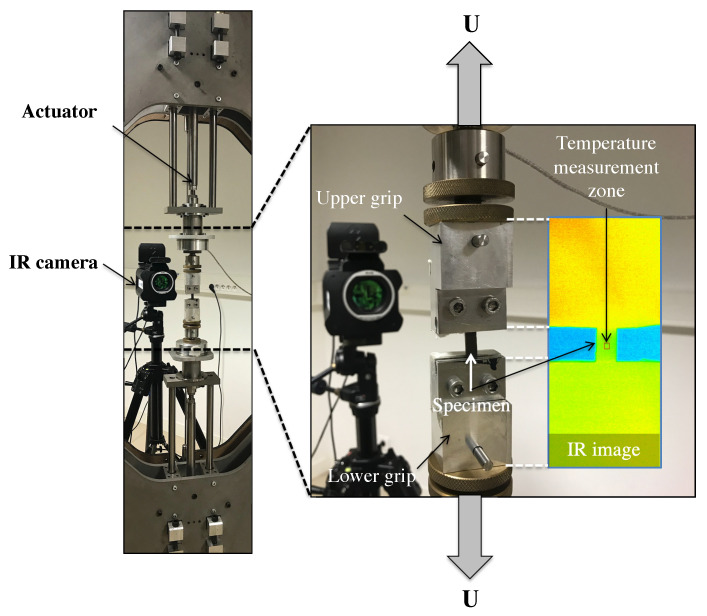
Typical experimental setup: the specimen is stretched symmetrically with a home-made biaxial testing machine, the temperature field is measured with an infrared camera.

The experimental setups are briefly recalled in Table 2. It should be noted that the loading conditions differ from one study to another, depending on the considered application, but lead to thermodynamical cycles from which energy balance can be carried out. Generally, the stretch is calculated from the initial length of the virgin specimens and is not corrected while a permanent deformation can appear and grow as the cycles follow each other. The temperature was measured with infrared cameras, which were switched on several hours before testing in order to ensure their internal temperature to be stabilized. The calibration of camera detectors was performed with a black body using a Non-Uniformity Correction (NUC) procedure. A typical experiment setup is given in Figure 1, which enabled us to stretch the specimens symmetrically. All the specimens tested had cylindrical ends that avoid any slippage with the grips and makes accurate the determination of the initial length.

### Remark on the Temperature Measurement

If the specimen is stretched by the displacement of only one jaw, which means that the initial temperature measurement area is shifted in the tensile direction, the temperature field must be initially homogeneous in order to calculate the heat sources. However, most of the testing machines and in particular the hydraulic ones lead to a thermal gradient in the specimen because the jaws are not at the same temperature. Therefore, a thermal camera has to be used in order to track the measurement area in the thermal images (see for instance [21]). If the specimen is stretched by moving the two jaws symmetrically, the initial temperature measurement area does not move and only one spot measurement with, for example, a pyrometer is possible, which reduces drastically the thermal measurement cost.

## 4. Results and Discussion

**Figure 2:**
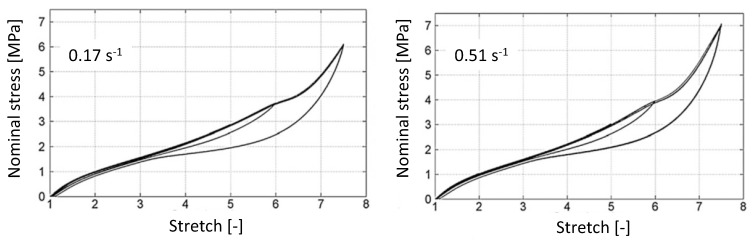
Mechanical responses of the unfilled NR obtained at ±0.17s${}^{-1}$ (on the left hand side) and ±0.51s${}^{-1}$ (on the right hand side), extracted from [21].

Figure 2 presents the mechanical responses of the unfilled NR obtained at ±0.17s${}^{-1}$ (on the left hand side) and ±0.51s${}^{-1}$ (on the right hand side) in terms of the nominal stress versus the stretch. They are extracted from [21]. For the two loading rates, no stress softening is observed between cycles and the loading rate has no effect on the stiffness expect for the highest stretch at $\lambda_{4}=7.5$ that induces the highest crystallinity. In this case, the highest loading rate leads to the highest stiffness. For cycles at $\lambda_{1}=2$ and $\lambda_{2}=5$, no hysteresis loop is observed. For cycles at $\lambda_{3}=6$, a hysteresis loop forms, which closes at a stretch equal to 3, which is close to the stretch at which crystallite melting is assumed to be complete. It should be noted that the crystallization onset is about 4, meaning that the crystallinity is not high enough to form a hysteresis loop. For cycles at $\lambda_{4}=7.5$, a plateau is observed from λ=6 on, followed by a stress increase that is higher at the highest loading rate, as previously observed and explained in [55,56]. As for the previous cycles at $\lambda_{3}=6$, the hysteresis loop closes at around λ=3.

**Figure 3:**
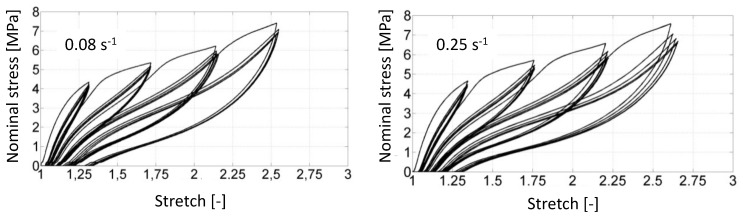
Mechanical responses of the TPU obtained at ±0.08s${}^{-1}$ (on the left hand side) and ±0.25s${}^{-1}$ (on the right hand side), extracted from [45].

Figure 3 presents the mechanical responses of the TPU obtained at ±0.08s${}^{-1}$ (on the left hand side) and ±0.25s${}^{-1}$ (on the right hand side), extracted from [45]. The mechanical responses exhibit softening, residual stretch and hysteresis, which was widely investigated in the literature by [57,58,59,60] and is similar to that observed in filled rubbers. It should be noted that the loading rate does not affect significantly the stiffness, which is a less intuitive result when considering that a hysteresis loop is mainly due to viscosity. This is further discussed in [45].

**Figure 4:**
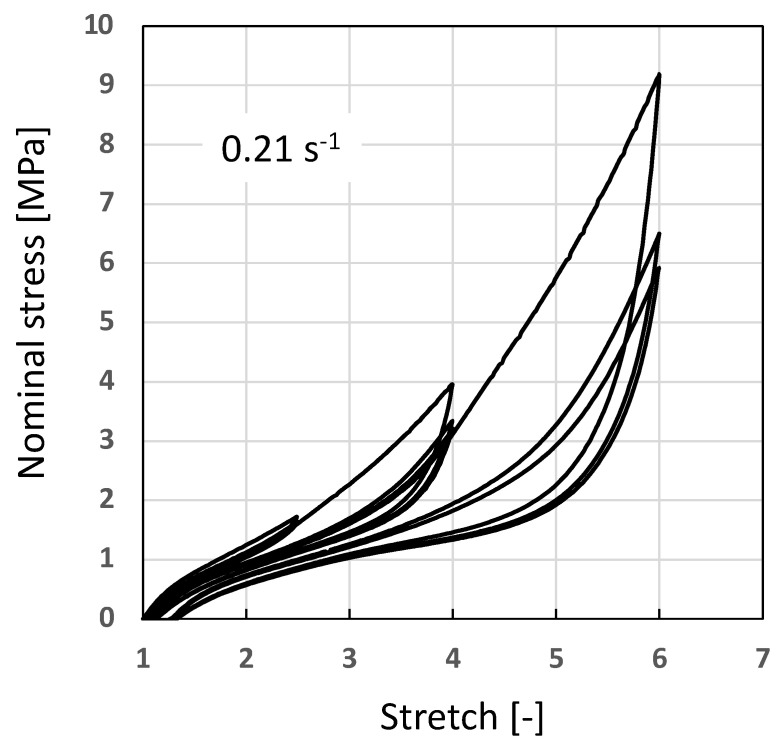
Mechanical response of the F-NR obtained at ±0.21s${}^{-1}$, extracted from [47].

Figure 4 presents the mechanical response of the F-NR obtained at ±0.21s${}^{-1}$, extracted from [47]. As expected, adding fillers to a natural rubber induces a hysteresis loop, this is the reason why it is observed in the mechanical response even at a stretch inferior to the crystallization onset. Furthermore, a stress softening is observed as well as a permanent set. In the case of a filled crystallizing rubber, a typical point is to define what is the part of the energy converted into heat and the one stored reversibly in one thermodynamical cycle. Obviously, this has a consequence on the self-heating and on the energy available for damaging the material, typically discussed in studies dealing with fatigue and fracture mechanics in elastomers.

The hysteresis loop observed in the mechanical responses of these three materials is due to the different possible contributions recalled in the introduction section. The aim of the following is to identify these contributions. For that purpose, the methodology described in Section 2 has been applied to each material:

- First, the heat source *S* has been calculated from Equation (3) for each stabilized cycle at different maximum stretches (these cycles are considered as thermodynamical ones),
- Second, $P_{hyst}^{cycle}$ and ${\tilde{D}}_{int}$ have been determined from Equations (2) and (4) respectively,
- Third, the $\gamma_{se}$ ratio has been calculated from Equation (6).

**Figure 5:**
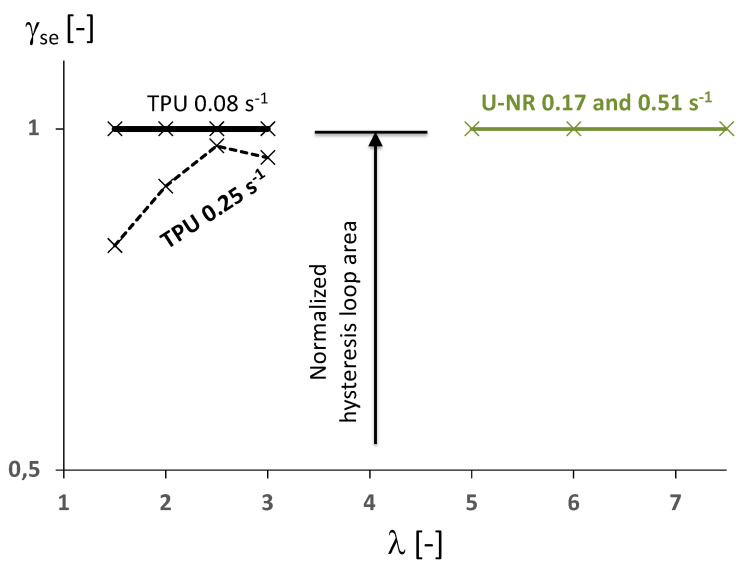
Energy characterization in terms of $\gamma_{se}$ versus stretch for the U-NR at 0.17 and 0.51 s${}^{-1}$ (in green) and the TPU (in black).

In the following, $\gamma_{se}$ is first plotted versus stretch for the unfilled NR and the TPU in Figure 5, which provides the first comparison of rubber compounds with respect to their energy behavior. We recall here that the higher the value of $\gamma_{se}$, the higher the energy storage capacity of the material. If $\gamma_{se}=1$, then no intrinsic dissipation is involved in the hysteresis loop, that is, no mechanical energy is converted into heat and 100% of the normalized area of the hysteresis loop is energy stored and released during the cycle. The $\gamma_{se}$—stretch relationship for the unfilled NR is given by the green curve in the diagram and was found close to 1, whatever the loading rate and the stretch level once the material is crystallizing ($\lambda >\lambda_{c}\approx 4$). For stretches inferior to the crystallization onset, no hysteresis loop forms. This illustrates, as demonstrated in [22], that all the energy involved in the hysteresis loop is stored by the crystallization processes and fully released during the crystallite melting. It can be therefore seen as a “cold energy”. For the TPU material at the strain rate of ±0.25s${}^{-1}$, $\gamma_{se}$ is about 0.8 at a stretch of 1.5 and increases with stretch. It is close to 1 for stretches from 2.5 on. The energy involved in the hysteresis loop is therefore almost not converted into heat and the higher the stretch level the higher the relative energy stored in the material. This strong similitude with the unfilled natural rubber is explained by the fact that the TPU considered here crystallizes under tension, but also by the fact that the multi-phase nature of TPU induces strong self-organization as suggested in [27] and regardless of whether it crystallizes or not as shown in [61]. It should be noted that for a strain rate of four times lower, the ratio is close to 1 whatever the stretch applied.

**Figure 6:**
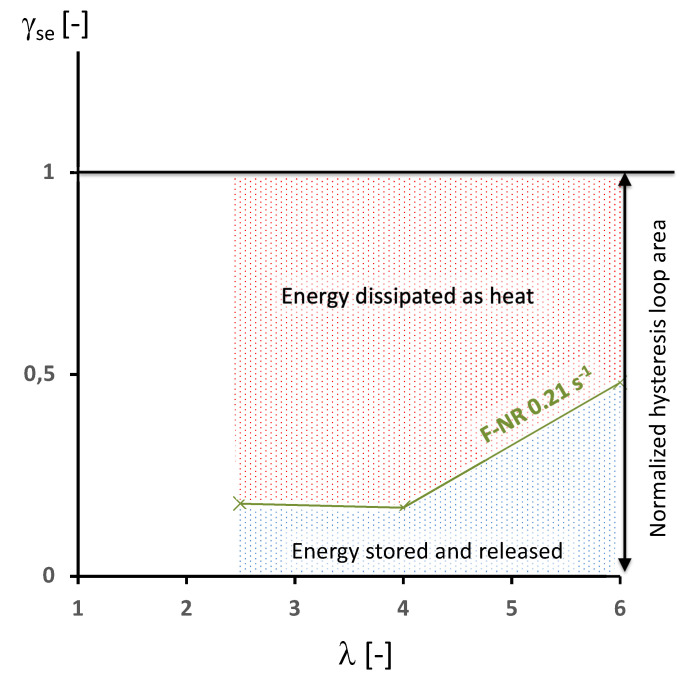
Energy characterization in terms of $\gamma_{se}$ versus stretch for the F-NR at 0.21 s${}^{-1}$.

Figure 6 gives $\gamma_{se}$ versus stretch for the filled NR stretched at the rate of the same order of magnitude as the lowest rate applied to the unfilled NR. It was observed that about 80% of the energy involved in the hysteresis loop is dissipated as heat at the lowest stretches applied (maximum 4, which induces a very low crystallinity). This means that adding fillers does not only induce viscosity but enables the material to store a part of the mechanical energy without converting it into heat, mechanical energy that is not used to damage and self-heat the material (this energy storage effect of the filler network was also highlighted in the case of carbon black filled acrylonitrile rubber (see [46] for further information)). When the material is crystallizing (when stretch at λ=6), the relative energy stored strongly increases from 20% of the hysteresis loop area at λ=4 to 50% ($\gamma_{se}=0.5$) at λ=6. As industrial natural rubbers are filled, comparing their energy behavior amounts to comparing the area under the red curve in the diagram. Typically, the lower this area in the deformation domain defined by the industrial application, the higher the self-heating and energy used to damage the material and the lower the elastic energy stored reversibly. From an elastocaloric point of view, identifying the relative contribution of the intrinsic dissipation during one cycle is of paramount importance as it opposes the heat absorption during the unloading and consequently it reduces the cooling.

## 5. Conclusions

This paper presents a simple and fast approach to determining the energy behavior of elastomers from cyclic uniaxial tensile loadings and temperature measurements. The temperature measurement is used to reconstruct the heat source, that is, the calorimetric response, from the 0D formulation of the heat diffusion equation. Based on the mechanical and calorimetric responses, a ratio $\gamma_{se}$ is calculated to relativise the energy stored and released at each cycle. Results show that viscosity is not systematically the preponderant contribution to the hysteresis loop, whatever the elastomer considered, filled and unfilled, crystallizing or not: the mechanical energy brought to the material is not entirely dissipated into heat and can be mainly used by the material to change its microstructure. Therefore, $\gamma_{se}$ assesses the ability of the material to store mechanical energy through reversible microstructure changes, energy that is not dissipated as heat and used to damage the material, for example, to propagate cracks. The results obtained enable us to characterize and to compare different materials according to their energetic performance. This study thus proposes an approach well adapted to the design of elastomer parts and opens many perspectives to discriminate, in an accelerated way, the factors affecting these energetic performances, at the first order are obviously the formulation, the aging and the mechanical loading. This makes it a very promising tool for further investigating the thermomechanical behavior of rubbers and for validating and enriching physical thermodynamical models. 

## Figures and Tables

**Table 1:** Chemical composition in parts per hundred rubber (phr).

Ingredient	U-NR [21]	F-NR [47]
Natural rubber NR	100	100
Carbon black	0	20–30
Antioxidant	1.9	2–4
Stearic acid	2	2
Zinc oxide ZnO	2.5	10
Accelerator	1.6	2–4
Sulfur	1.6	1.5

**Table 2:** Summary of the experiments performed.

Materials			
Reference	U-NR	F-NR	TPU
	[21]	[47]	[45]
Filler type	-	CB	-
and amount (phr)	0	20–30	
Crystallizable under strain	Yes	Yes	Yes
Specimen geometry (mm): Width × Length × thickness	5 × 10 × 1.4	10 × 24 × 2	9 × 20 × 5
Testing machine	Instron 5543, one moving grip	Homemade biaxial tensile machine, symmetric loading	Instron 5543, one moving grip
Mechanical loading	3 cycles at λ=2, 5, 6 and 7.5	3 cycles at λ=2.5, 4 and 6	5 cycles at λ= 1.5, 2, 2.5 and 3
Constant loading rate (mm/min)/strain rate (s${}^{-1}$)	±100 and ±300/±0.17 and ±0.51	±300/±0.21	±100 and ±300/±0.08 and ±0.25
Infrared camera, resolution	Cedip Jade III, 320 × 240 px	FLIR X6540sc, 640 × 512 px	FLIR X6540sc, 640 × 512 px
Motion compensation technique	Yes	No	Yes

## Data Availability

Data can be found in [21,45,47].

## Abbreviations

The following abbreviations are used in this manuscript:

CB	Carbon Black
F-NR	Filled Natural Rubber
F-NBR	Filled Nitrile Butadiene Rubber (acrylonitrile rubber)
phr	part per hundred of rubber in weight
NR	Natural Rubber
SIC	Strain-Induced Crystallization
TPU	Thermoplastic PolyUrethane
U-NR	Unfilled Natural Rubber
